# Use of a Serum With the Infinite Glow® Device Improves Signs of Facial Aging: A Split-Face Case Report Using VISIA Analysis

**DOI:** 10.7759/cureus.111375

**Published:** 2026-06-23

**Authors:** Dominique Rodríguez, Manuel Rodríguez, Christopher Andersen

**Affiliations:** 1 General Medicine, Dermatological Orlandi Clinic, Santiago, CHL; 2 Dermatology, Dermatological Orlandi Clinic, Santiago, CHL; 3 Dentistry, Nuva Group Spa, Santiago, CHL

**Keywords:** anti-aging medicine, device development, facial cosmetic, skin aging, split-face

## Abstract

An increase in wrinkles, laxity, and dyschromia characterizes facial-skin aging. The skin is a natural barrier that prevents or limits the entry of external therapeutic compounds to the body. Enhancing the efficacy of transdermal delivery of anti-aging compounds is a key therapeutic strategy. The study aimed to evaluate the efficacy and safety of a combined therapy that included a topical anti-aging serum and a multi-technology facial device designed to improve transdermal absorption. A 41-year-old woman with moderate facial aging was treated using a split-face design for 12 weeks. Every day, the left hemiface received a hyaluronic acid-niacinamide-Matrixyl serum. In contrast, the right hemiface received the same serum plus application of a multi-therapy device that delivered red light (630 nm), galvanic current, micro-vibration, and controlled heat. Skin changes were assessed using the VISIA Skin Analysis System (Canfield Scientific, Parsippany, New Jersey) at baseline, 6, and 12 weeks. Both hemifaces showed improvement in skin quality parameters (wrinkles, texture, and UV spots). TruSkin age decreased by five years on the treated side with the device + serum and by one year on the serum-only side. Greater improvement in wrinkle reduction was observed on the device-treated side. No adverse events were reported. As a preliminary observation, combined therapy using a topical serum and a multimodal device appears safe and attenuates aging effects, particularly in reducing wrinkles. Further studies are needed to confirm these findings.

## Introduction

Skin aging is influenced by multiple key factors that may accelerate its natural progression, including hormonal changes, genetic alterations, cellular oxidative stress, and exposure to UV radiation. These factors contribute to structural and functional changes in the skin, resulting in clinically visible features such as wrinkles, laxity, reduced elasticity, and depigmentation. The facial skin is particularly susceptible to aging-related changes, which has driven the development of a wide range of cosmetic and therapeutic interventions aimed at attenuating the effects of the aging process [1].

Transdermal drug absorption is a complex process primarily governed by the barrier function of the stratum corneum, the outer layer of the skin. The absorption efficiency depends not only on the physicochemical properties of the drug, such as molecular weight, lipophilicity, solubility, and ionization state, but also on factors related to the skin itself, such as hydration status, thickness of the stratum corneum, anatomical site, age, and skin integrity [2]. Furthermore, local blood flow and skin temperature can influence the diffusion and distribution of topically applied compounds [3]. A key factor in non-invasive (injection-free) skin rejuvenation treatments is enhancing the transdermal absorption of anti-aging molecules.

Regarding anti-aging molecules, in this study, we highlight the properties of 1-hyaluronic acid that enhances skin hydration and turgor through its remarkable water-retaining capacity and its role in extracellular matrix homeostasis [4]; 2-niacinamide (B3 vitamin) that improves epidermal barrier function, reduces transepidermal water loss, and exerts antioxidant and anti-inflammatory effects [5]; and 3-matrikines, which act as signaling peptides that stimulate fibroblast dermal remodeling and wrinkle reduction [6].

The aim of this clinical case was to preliminarily evaluate the anti-aging effect and safety of a combined therapy composed of a topical serum (with niacinamide, hyaluronic acid, and a mix of matrikines) and a multi-modal device designed to promote transdermal absorption using galvanic current, red light, soft micro-vibration, and controlled heat in a woman with moderate facial aging signs.

## Case presentation

A 41-year-old woman presenting with moderate signs of facial aging was evaluated according to the Glogau record [7]. Clinical examination revealed under-eye wrinkles, crow's feet, nasolabial folds, solar lentigines, skin dullness, and reduced skin hydration corresponding to Glogau photodamage type II/III. The patient had not experienced any changes in anti-conceptive hormone therapy in the last six months and had no history of prior aesthetic treatment or procedure, smoking, or dermatological diseases. The patient was informed and trained about the procedure before its execution and signed an informed consent.

The study has a split-face design. Every evening before going to bed, the patient applied the serum to her left hemiface and the serum and the Infinite Glow device to her right hemiface every day for three months. The device was applied for three minutes for each region: 1-front, 2-around the eye and cheek, and 3-in the chin. Patient follow-up was performed at 6 and 12 weeks of treatment. The study design was explained to the patient, emphasizing the importance of caring for both sides of her face equally, for example, by avoiding sun exposure with sunscreens.

The serum is a solution based on hyaluronic acid of high (2.5%) and low (2.5%) molecular weights, 3% Matrixyl® (palmitoyl pentapeptide-4 and related matrikines), and 5% niacinamide.

**Figure 1:**
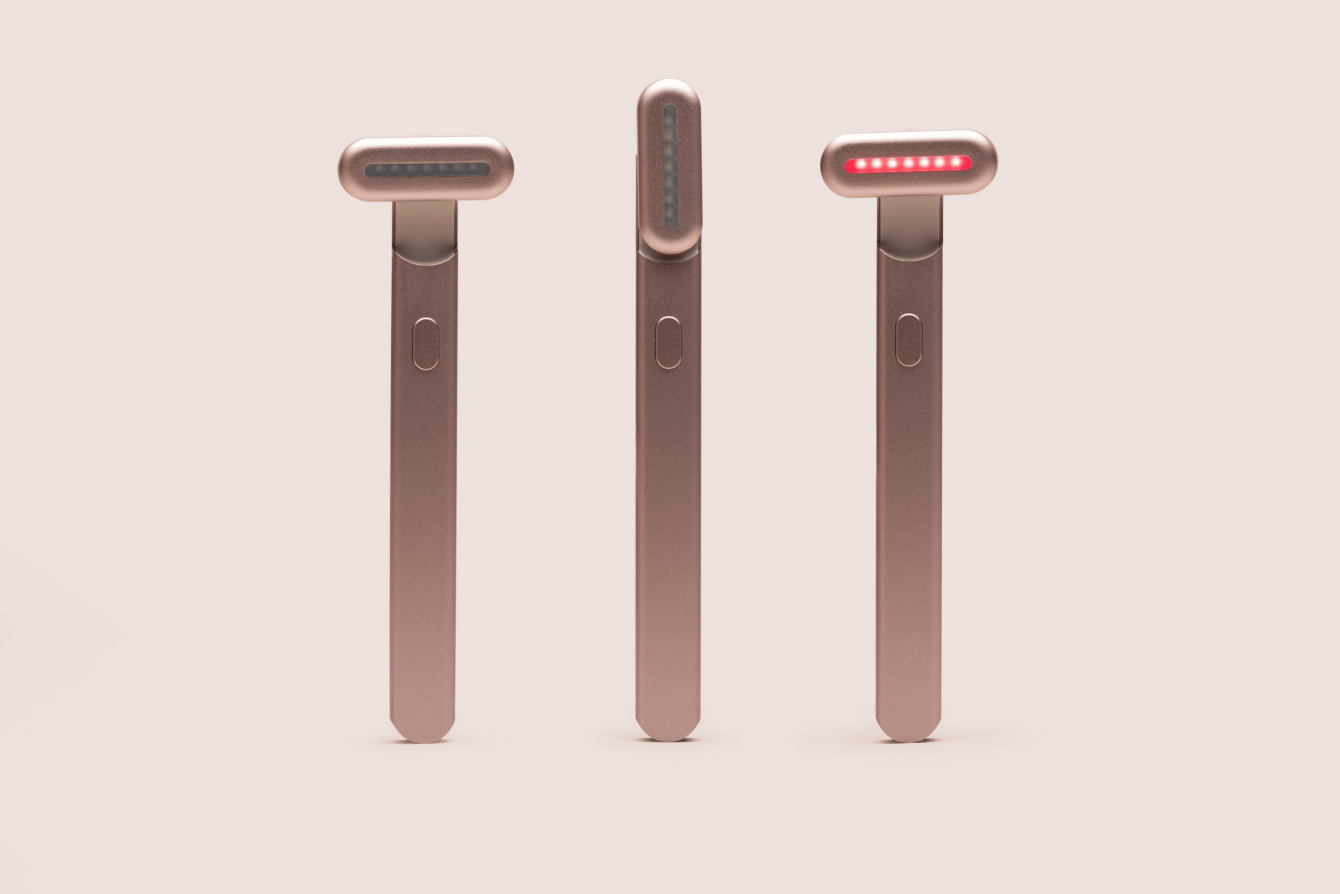
Representative image of the Infinite Glow(R) device in on and off mode. Original image by the authors.

Infinite Glow® (developed by Nuva Group Spa) is a portable facial device composed of four skin stimulation technologies: (i) red light radiation with a wavelength of 630 nm; (ii) low-intensity galvanic current generation; (iii) a micro-vibration and facial massage system; and finally, (iv) controlled therapeutic heat, designed to provide a sensation of comfort on the skin, promoting improved local blood circulation (Figure 1). The device has been recognized as a utility model by the Instituto Nacional de Propiedad Intelectual (INAPI) under Nº20240112 [8].

The efficacy of the Infinite Glow device + serum was evaluated using the VISIA Skin Analysis System (Canfield Scientific, Parsippany, New Jersey). This camera-based device rotates around the patient’s face to capture high-resolution, multispectral photos for evaluating skin health [9]. We evaluate the levels of wrinkles, texture, spots, pores, UV spots, brown spots, red areas, porphyrins, and TruSkin age parameters in comparison with a database of 250,000 persons of different ages [9]. For each parameter, the software generates a percentile score by comparing the patient’s results with an age-, sex-, and skin-type-matched reference database. Percentiles represent the relative position of the individual within the reference population. 

To minimize the effect of baseline facial asymmetry, each hemiface was analyzed longitudinally, comparing post-treatment values with its own baseline measurements. We obtained baseline values of 41 and 44 for TruSkin Age on the right and left hemifaces, respectively. After three months of treatment, this parameter decreased by 5 and 2 years in the right and left hemifaces, respectively. The main parameters that contributed to these results are spots, wrinkles, and texture. UV spots (expressed in percentiles relative to the age-matched reference population) increased at 6 weeks in the right hemiface (from 58 to 86) and in the left hemiface (from 72 to 86), and the values remained almost the same at 12 weeks, 87 vs. 82 left and right hemifaces, respectively. These data suggest an improvement in the UV spots percentile during treatment. Regarding wrinkles, we observed a reduction of wrinkle expression in the right hemiface, expressed in percentiles (T0: 34, T6w: 88, and T12w: 64) and in the left hemiface (T0: 59, T6w: 88, and T12w: 61). As we showed in the spots parameter, the texture value (expressed in percentile) has the same tendency, for the right hemiface (T0: 21, T6w: 40, and T12w: 17) and the left hemiface (T0: 16, T6w: 40, and T12w: 15). Data are summarized in Table 1.

**Table 1:** Effect of aesthetic treatment measured by the VISIA parameters (wrinkles, UV spots, and texture) expressed as percentile values relative to an age-matched reference population.

VISIA^(R)^ parameters	Hemifaces	Percentiles		
		Baseline	6 weeks	12 weeks
Wrinkles	Right	34	88	64
	Left	59	88	61
UV spots	Right	58	86	82
	Left	72	86	87
Texture	Right	21	40	17
	Left	16	40	15

**Figure 2:**
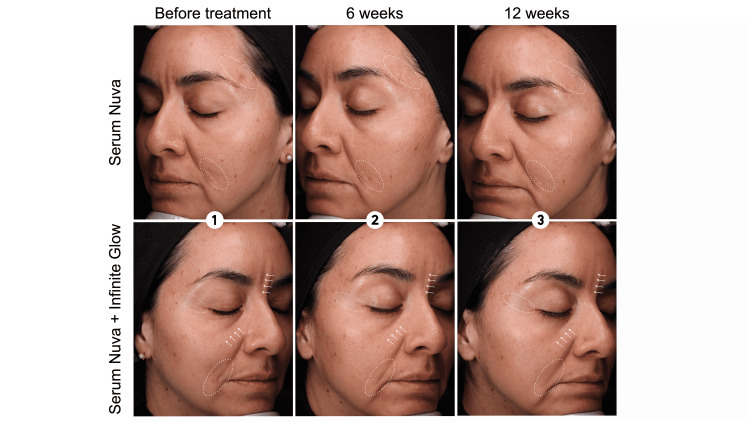
Effect of aesthetic treatment in a split-face description. White ovals with dashed lines indicate changes in skin color, while white arrows highlight the attenuation of skin wrinkles.

In Figure 2, we outlined the visible effects of the aesthetic treatment as white ovals with dashed lines, indicating changes in skin color, while arrows highlight the attenuation of skin wrinkles. No adverse event was reported.

## Discussion

We observed a beneficial effect on both sides of the face, that is, a reduction in skin age, which was more evident at 6 weeks of treatment. Regarding the effect of the Infinite Glow device, we observed a significant benefit in reducing wrinkles. These results may be explained due to (i) galvanic current facilitates transdermal delivery of bioactive compounds through iontophoresis, improving the penetration and effectiveness of topical agents [2,10]; (ii) red light radiation induces photobiomodulation, stimulating mitochondrial activity and promoting collagen synthesis via fibroblast activation [11]; (iii) micro-vibration provides mechanical stimulation that may enhance microcirculation, lymphatic drainage, and mechanotransduction pathways involved in extracellular matrix remodeling [12]; and finally, controlled thermal stimulation promotes vasodilation, increasing local blood flow and supporting tissue metabolism. Overall, these technologies act synergistically, primarily as adjunctive strategies to improve skin quality and enhance the effects of topical treatments rather than as standalone anti-aging interventions.

An important objective for us was to evaluate the device's safety, given its innovative design that combines four therapies into a single device. The safety and efficacy of the combined therapy encourage further studies to confirm these results, particularly the long-term persistence of the rejuvenation effect.

## Conclusions

A combined non-invasive approach integrating a bioactive topical serum with a multimodal device showed improvements in key skin aging indicators, including wrinkle attenuation, at 6 weeks of treatment in this case report. These preliminary findings support the concept that facilitating transdermal delivery and stimulating complementary biological pathways may synergistically improve skin rejuvenation outcomes. Further controlled studies with larger populations and longer observation periods are warranted to validate these findings.
